# Association of Legume Intake with Incident Hyperuricemia: A Prospective Cohort Study in Shanghai Adult Residents

**DOI:** 10.3390/nu18091355

**Published:** 2026-04-24

**Authors:** Xiaoli Xu, Mengru He, Na Wang, Xing Liu, Minqi Wei, Yonggen Jiang, Qian Peng, Jianhua Shi, Dandan He, Genming Zhao

**Affiliations:** 1 Minhang District Center for Disease Control and Prevention (Minhang District Institute of Health Supervision), Shanghai 201108, China; lisa861227@126.com (X.X.);; 2 School of Public Health, Fudan University, Shanghai 200032, China; 3 Songjiang District Center for Disease Control and Prevention (Songjiang District Institute of Health Supervision), Shanghai 201600, China; 4 Jiading District Center for Disease Control and Prevention (Jiading District Institute of Health Supervision), Shanghai 201800, China; 5 Xuhui District Center for Disease Control and Prevention (Xuhui District Institute of Health Supervision), Shanghai 200237, China

**Keywords:** legumes, hyperuricemia, cohort study, Shanghai adult, Chinese population

## Abstract

**Objective:** To evaluate the relationship between legume intake and incident hyperuricemia among Chinese adults using large-scale prospective cohort data. **Methods:** Baseline and follow-up information from the Shanghai Suburban Adult Cohort and Biobank (SSACB) were used to assess diet and hyperuricemia incidence [serum uric acid (SUA) ≥ 420 μmol/L in males and ≥ 360 μmol/L in females]. A Food Frequency Questionnaire (FFQ) covering 29 food categories quantified food consumption during the previous 12 months. Legume intake was calculated by multiplying the reported consumption of each item by (1 − water content), and participants were classified into tertiles with the lowest third (Q1) as the reference. Cox proportional-hazards models estimated hazard ratios (HRs) and 95% confidence intervals (*CIs*), and restricted cubic splines (RCSs) with three knots (10th, 50th, and 90th percentiles) visualized the dose–response relation. **Results:** Among 43,371 participants, 1456 new cases of hyperuricemia were documented over 225,002.40 person-years (incidence density 6.47/1000 person-years; 95% *CI* 6.14–6.80). Incidence density decreased with higher legume intake: each 1 g/day increment was associated with a 2% lower risk (HR 0.98; 95% *CI* 0.97–0.99; *p* < 0.05). Compared with Q1, the highest tertile (Q3) showed a 26% risk reduction in the fully adjusted model (HR 0.74; 95% *CI* 0.64–0.86; *p* < 0.05). RCS revealed a significant nonlinear relationship (*p*-overall < 0.001, *p*-nonlinear = 0.0013), with the significant benefit in risk observed at 6–28 g/day. **Conclusions:** Legume intake is nonlinearly and inversely associated with hyperuricemia risk among Shanghai suburban adults. Given that the current low median intake, comprehensive strategies are needed to rationally adjust the dietary structure, improve legume intake, and provide sustainable development strategies for effective prevention and control of hyperuricemia.

## 1. Introduction

Legumes are rich in 35–40% protein, and their soybean protein includes nine kinds of essential amino acids needed by the human body, which is considered to be a complete high-quality protein [1,2]. Its ratio of amino acids is close to the human body’s demand, making it a highly important plant-based source of dietary protein for humans, and it has become a sustainable and healthy nutritional alternative to meat [3]. At the same time, legumes are also rich in folic acid, iron, zinc, potassium, magnesium and other minerals and bioactive components [4]. In multinational dietary guidelines, legumes are recommended as one of the food categories [5,6]. As one of the foods with low Glycemic Index (GI) [7], legumes have been confirmed by many prospective studies at home and abroad, and the increase in their intake is related to the reduction in all-cause risk, stroke mortality, vascular events and some cancers [8,9,10,11,12].

The intake of legumes is beneficial to human health, and soybean protein and peptides have functions related to chronic diseases, including anti-obesity, anti-diabetes, anti-cardiovascular disease regulation and anti-cancer activity [13]. Most epidemiological data show that legume food intake is inversely related to uric acid level [14]. The equilibrium of circulating uric acid levels hinges on the interplay between production and excretion. The kidney plays a dominant role in uric acid excretion, responsible for approximately 70% of the daily produced uric acid, while the remaining 30% is excreted by the intestine [15]. The medical point of view is that this delicate balance is orchestrated by the functions of uric acid transporters across various epithelial tissues and cell types, and hyperuricemia develops when uric acid production surpasses its excretion [16].

Globally, from 2000 to 2023, the prevalence of hyperuricemia in women increased from 6.7% to 11.2%, and that in men increased from 12.3% to 18.6% [17]. A cross-sectional survey from 31 provinces of the Chinese mainland has shown that the weighted prevalence of hyperuricemia among adults in China was as high as 17.7% [18]. It can be seen that the disease burden of hyperuricemia is increasing, especially in high-income and urban settings [17], which is considered the key risk factor for gout and other diseases [19,20] and has become an important public health problem in the world.

Therefore, studying the association between the intake of legumes and the incidence of hyperuricemia in adults will help to understand the role of legume food intake in preventing hyperuricemia, so as to improve people’s health, which has important scientific value and public health significance. Although the weighted prevalence of hyperuricemia remains high among Chinese adults, with coastal cities including Shanghai showing more pronounced prevalence compared to inland and rural areas [18], prospective cohort studies focusing on adult populations in Shanghai are extremely scarce [21]. As a highly urbanized coastal metropolis in China, Shanghai is confronted with the dual challenges of an increasing disease burden of hyperuricemia and an ongoing transformation of dietary structure. Therefore, we conducted this study utilizing large-scale prospective cohort data, including an adult cohort and a biological bank in Shanghai suburbs, and proposed the following hypothesis: legume intake is inversely related to hyperuricemia incidence among adults in suburban Shanghai, with the association persisting after comprehensive adjustment for confounders such as sociodemographic, lifestyle, and physiological variables. The aim of this study is to explore the association between legume intake and hyperuricemia incidence in adults. These findings are expected to provide important scientific evidence for developing precision dietary intervention strategies suitable for the Chinese population, especially those in coastal cities.

## 2. Materials and Methods

### 2.1. Study Design and Participants

Established in 2016, the Shanghai Suburban Adult Cohort and Biobank (SSACB) recruited 69,116 residents aged between 20 and 74 by cluster random sampling, which is a prospective cohort study. The Ethical Review Committee of the School of Public Health, Fudan University, approved the protocol for the study (IRB approval number 2016-04-0586-S1, 2 September 2024), and participants provided written informed consent. This analysis used the SSACB, which has been reported in previous studies on different exposure–outcome pairs; the present manuscript addresses the association of the intake of legumes with hyperuricemia. In this analysis, we included baseline participants with complete questionnaire data and physical examination information. The association between the intake of legumes and hyperuricemia incidence was examined up to February 2023. The Ethical Review Committee of the Minhang District Center for Disease Control and Prevention approved the protocol for the analysis (IRB approval number EC-P-2025-012, 30 December 2025).

The questionnaire survey was conducted by trained professional technicians using standardized structured questionnaires, including: (1) sociological demographic characteristics such as age, gender, education level, marital status and retirement status; (2) smoking, alcohol drinking, diet, physical activity (PA), sleep and other lifestyle information; and (3) physiological information such as chronic disease history and cancer history. All questionnaires were recorded simultaneously, and 5% of the recordings were randomly selected by trained quality control staff after the investigation to ensure their reliability.

Physical examination was carried out by professional clinicians by standardized methods, which included anthropometric indicators such as height, weight, waist circumference (WC) and blood pressure. At the same time, blood samples were collected after fasting for 8 h (stored in the freezing room at −80°C), and biochemical tests were performed within 6 h. Each participant has a unique identification code, and their follow-up records are related to the Electronic Medical Record (EMR) System and the Cause-of-Death Surveillance System (CDSS). The coding standard of the International Classification of Diseases-10 (ICD-10) was adopted for disease diagnosis. Details of the SSACB have been reported previously [22,23].

**Figure 1:**
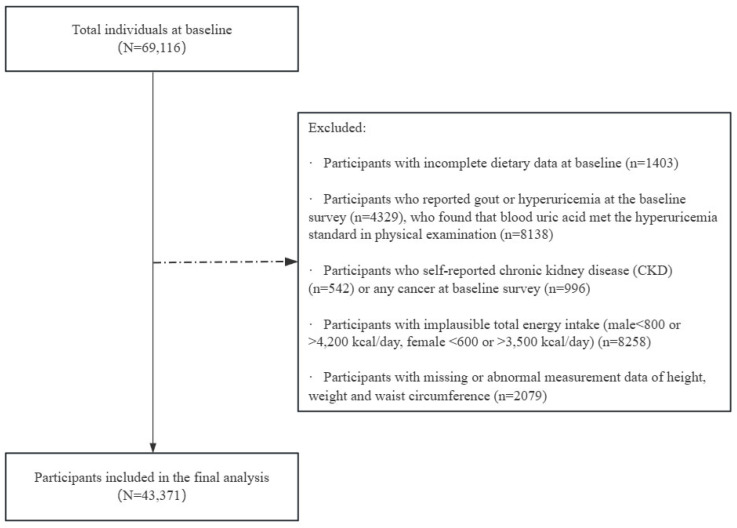
Flow of the participants for the current study.

We first excluded 1403 participants with incomplete dietary data, then 4329 subjects had reported gout or hyperuricemia at the baseline survey, 8138 subjects found that blood uric acid met the hyperuricemia standard in physical examination, 542 subjects with chronic kidney disease (CKD) and 996 subjects self-reported any cancer were also excluded. Among the remaining individuals, 8258 subjects with unreliable total energy intake (beyond the following range: 800–4200 kcal/day for males and 600–3500 kcal/day for females) and 2079 subjects with missing or abnormal anthropometric indicators (including height, weight and WC) were further excluded. Finally, 43,371 participants aged between 20 and 74 were included in this analysis (15,678 males and 27,693 females), as shown in Figure 1.

### 2.2. Assessment of the Intake of Legumes

Dietary data were obtained from the Food Frequency Questionnaire (FFQ). The questionnaire was used to evaluate participants’ food consumption. By asking participants “the frequency of legume food intake in the past 12 months and each intake”, the participants’ consumption information of legume food was obtained. Previous studies have confirmed that the FFQ is reasonable and effective in evaluating nutrient intake [24]. The FFQ is divided into eight frequency categories (never, not less than 1 time/month, 1–3 times/month, 1–3 or 4–6 times/week, and 1, 2 or more times/day). The intake of legumes is evaluated through two categories: soybean milk, tofu and its bean products (tofu, dried tofu, yuba, etc.). Due to the water content of various legumes being different (from 96.4% of soybean milk, 82.8% of tofu, 65.2% of dried tofu, to 10.2% of dried soybean), we also get the legumes’ intake by multiplying the consumption by (1 − the percentage of water content of food) [25,26,27]. Participants were divided into tertiles based on their legume food intake.

### 2.3. Ascertainment of Hyperuricemia

SUA was determined by automatic colorimetry. Based on the previous research, this study defined hyperuricemia as male SUA level (≥420 μmol/L), female SUA level (≥360 μmol/L) [28,29], and the ICD-10 was E79 in EMR, which is diagnosed by licensed physicians.

### 2.4. Assessment of Covariates

The covariates used in this study come from the baseline questionnaire and physical examination results, including:

(1) The adjusted sociological demographic characteristics cover age (years of age), gender (male/female), education level (primary school and below/junior high school/senior high school and above), marital status (unmarried/married/other circumstances including divorce, widowhood, etc.), and retirement (Yes/No).

(2) The adjusted lifestyle information includes smoking (Yes/No), alcohol drinking (Yes/No), diet and energy intake, PA level (low/medium/high level) [30], sleep time (<5/5–8/≥8 h), and body mass index (BMI) (<18.5/18.5–23.9/24–27.9/≥28.0 kg/m^2^) [31]. The diet and energy intake include cereals and potatoes, vegetables, mushrooms, fruits, red meat (the meat of mammals such as pigs, cows and sheep), white meat (including poultry such as chickens, ducks and geese, fish, shrimps, crabs and shellfish), milk and dairy products, eggs, nuts (peanuts, walnuts, almonds and melon seeds) and processed meat. According to the data from the FFQ, the intake status was calculated according to the China Food Composition Table [27].

(3) The adjusted physiological information includes diabetes [glycosylated hemoglobin A1C (HbA1C) ≥6.5% measured by physical examination, fasting plasma glucose (FPG) ≥7.0 mmol/L or previously diagnosed with diabetes] [32], hypertension (systolic blood pressure measured by physical examination ≥140 mmHg, diastolic blood pressure ≥90 mmHg, or previously diagnosed with hypertension) [33], dyslipidemia [total cholesterol measured by physical examination ≥6.20 mmol/L, high density lipoprotein cholesterol ≤1.00 mmol/L, low density lipoprotein cholesterol ≥4.10 mmol/L or triglyceride ≥2.30 mmol/L, or dyslipidemia diagnosed by previous doctor’s diagnosis] [34]; and self-reported chronic diseases such as coronary heart disease (CHD), chronic obstructive pulmonary disease (COPD), chronic bronchitis and asthma.

### 2.5. Statistical Analyses

Normality of continuous variables was examined with the Kolmogorov–Smirnov test. Normally distributed metrics were expressed as mean (standard deviation, SD) and compared by *t*-test or one-way analysis of variance (ANOVA); skewed variables were summarized as median (inter-quartile range, IQR) and compared by Kruskal–Wallis or Mann–Whitney U test. Categorical data were presented as n (%) and evaluated by the χ^2^ test. Incidence density of hyperuricemia was calculated as the number of incident cases divided by the total person-years of follow-up. Person-years of follow-up were calculated from the date at baseline to the mortality, the first occurrence, or the end of the study (28 February 2023), whichever came first.

Cox proportional-hazards models were used to estimate hazard ratios (HRs) and 95% confidence intervals (*CIs*) for the association between legume intake and hyperuricemia risk. Follow-up time (years) served as the time metric; the lowest tertile of legume intake (Q1) was the reference. Proportional-hazards assumptions were verified by the Schoenfeld individual test (all *p* > 0.05). Model 1 adjusted for sociological demographics: age (years), gender, educational level, marital status, and retirement. Model 2 additionally adjusted for lifestyle factors: smoking, alcohol drinking, PA level, sleep time, BMI, WC, dietary energy intake, and consumption of cereals and potatoes, vegetables, mushrooms, fruits, red meat, white meat, milk and dairy products, eggs, nuts, and processed meat. Model 3 further adjusted for physiological information: hypertension, CHD, diabetes, dyslipidaemia, COPD, chronic bronchitis, and asthma. Linear trend was evaluated by entering the median intake of each tertile as a continuous variable in the fully adjusted model.

A sensitivity analysis was repeated on Model 3 after (a) excluding participants who developed hyperuricemia within the first year, (b) within the first two years, and (c) excluding baseline participants under 40 years old to validate the robustness of the results. A subgroup analysis was performed across strata of age group (<60 or ≥60 years old), gender, smoking, alcohol drinking, BMI (<24 or ≥24 kg/m^2^), hypertension, CHD, diabetes, dyslipidaemia, and chronic bronchitis. Each model retained all covariates of Model 3 except the stratification variable itself. The dose–response relationship was visualized with restricted cubic splines (RCS) (3 knots: 10th, 50th, 90th percentiles) based on the fully adjusted Model 3. All analyses followed STROBE guidelines. Statistical procedures were carried out in SPSS 19.0 (SPSS Inc., Chicago, IL, USA) and R 4.3.2 (R Development Core Team, Vienna, Austria). Two-sided *p* < 0.05 was considered significant.

## 3. Results

### 3.1. Baseline Characteristics

The baseline characteristics of 43,371 participants according to the three categories of legume intake groups are presented in Table 1. Over a total of 225,002.40 person-years of follow-up (the median follow-up time was 5.59 years), we identified 1456 cases of new hyperuricemia, while the incidence density was 6.47/1000 person-years (95% *CIs*: 6.14–6.80/1000 person-years) in total. The incidence density decreased with the increase in the intake of legumes.

In general, 36.15% of the participants were male, and their median age was 58 years (50–65 years). Participants who consumed more legume food were more likely to be male, younger, with a higher education level, unmarried, unretired, smoking, drinking alcohol, with a higher physical activity level, a more suitable sleep time, higher dietary energy and more intake of other foods (except cereals and potatoes and vegetables), while they were unlikely to have a history of hypertension, chronic bronchitis and asthma.

### 3.2. Associations of the Intake of Legumes with Hyperuricemia

Table 2 shows the associations between the intake of legumes and the risk of hyperuricemia. Each 1 g/day increment was associated with a 2% lower risk (HR: 0.98, 95% *CI*: 0.97, 0.99; *p*-value < 0.05). Moreover, compared to the lowest tertile (Q1), the highest tertile (Q3) was linked to a significant 26% reduction in risk (HR: 0.74, 95% *CI*: 0.64, 0.86; *p*-value < 0.05). These patterns were statistically significant (*p* for trend < 0.001) across all models, as shown in Table 2.

### 3.3. Sensitivity Analysis

We conducted a sensitivity analysis to verify the robustness of the results. When participants diagnosed with hyperuricemia in the first or the first two years after the baseline survey were excluded, the results were similar. The results were similar when participants aged under 40 years old in the baseline survey were excluded. In any of the above sensitivity analyses, the results have not changed fundamentally. Tests of trends were statistically significant in each model (*p* for trend < 0.001). Details are shown in Table 3.

### 3.4. Subgroup Analysis

**Figure 2:**
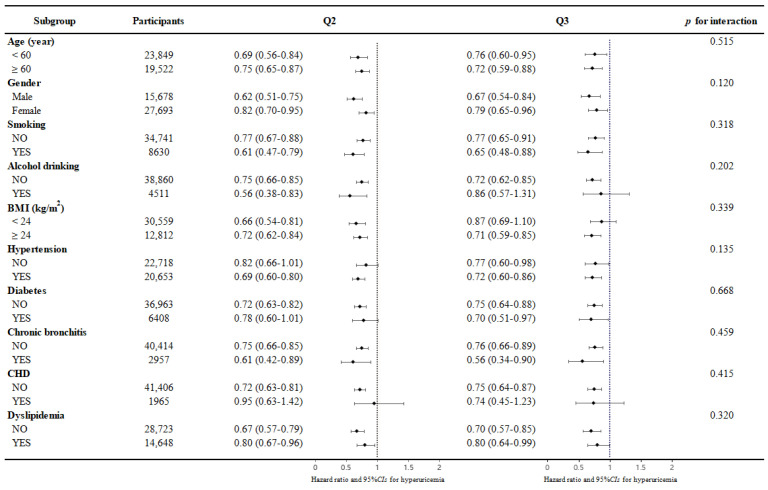
Association between the intake of legumes and hyperuricemia by subgroups of age, gender, smoking, alcohol drinking, BMI group, and the history of chronic diseases (including hypertension, CHD, diabetes, dyslipidemia, and chronic bronchitis). The model was adjusted for age, gender, educational levels, marital status, retirement, smoking, alcohol drinking, PA levels, sleep time, BMI, WC, dietary energy intakes, consumption of cereals and potatoes, vegetables, mushrooms, fruits, red meat, white meat, milk and dairy products, eggs, processed meats, and physiological information (hypertension, CHD, diabetes, dyslipidemia, COPD, chronic bronchitis and asthma). In each subgroup analysis, adjustments were made for all previously mentioned covariates, excluding the specific covariate under investigation. Abbreviations: PA, physical activity; BMI, body mass index; WC, waist circumference; CHD, coronary heart disease; COPD, chronic obstructive pulmonary disease.

Subsequently, according to age group (<60, ≥60 years old), gender, whether smoking, alcohol drinking, and BMI group (<24 kg/m^2^, ≥24 kg/m^2^) and whether they had chronic diseases such as hypertension, CHD, diabetes, dyslipidemia, and chronic bronchitis, we conducted a subgroup analysis. Figure 2 shows the association between the legume intake group and hyperuricemia events in each subgroup after adjusting for all the covariates except itself. No significant interaction was found between the intake of legumes and all the above (all *p*-values for interaction > 0.05).

### 3.5. Dose–Response Analysis

**Figure 3:**
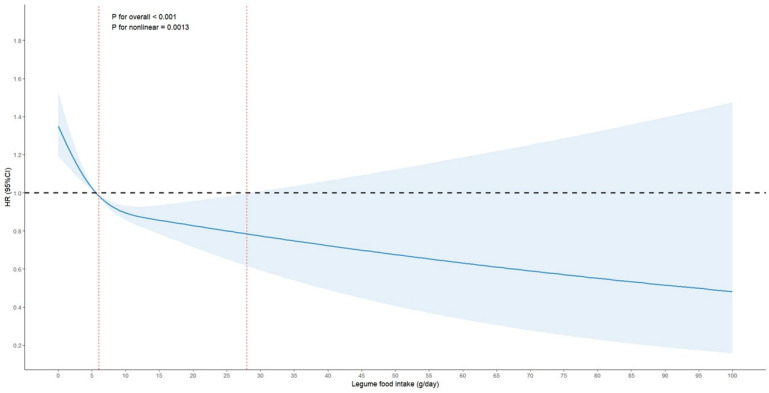
Association of the intake of legumes with hyperuricemia in RCS models. The model was adjusted for age, gender, educational levels, marital status, retirement, smoking, alcohol drinking, PA levels, sleep time, BMI, WC, dietary energy intakes, consumption of cereals and potatoes, vegetables, mushrooms, fruits, red meat, white meat, milk and dairy products, eggs, processed meats, and physiological information (hypertension, CHD, diabetes, dyslipidemia, COPD, chronic bronchitis and asthma). The HR is represented, with the point estimates (denoted by the blue line) and the corresponding 95% *CIs* (enclosed in a light blue shaded region), which were derived utilizing Cox proportional-hazards regression models that incorporated RCS with 3 knots set at the percentiles of 10th, 50th and 90th. The two vertical red dashed lines indicate the legume intake levels at which the lower and upper 95% confidence intervals of the HR first intersect the reference HR = 1.0, denoting the range of intake associated with a statistically significant change in risk. Abbreviations: RCS, restricted cubic splines; PA, physical activity; BMI, body mass index; WC, waist circumference; CHD, coronary heart disease; COPD, chronic obstructive pulmonary disease.

After careful consideration according to the intake of the legume group, we chose the RCS model of three knots (10th, 50th and 90th percentiles) and set the reference value as the lowest value. The results of the fully adjusted RCS model showed that there was a significant nonlinear relationship between the intake of legumes and the risk of hyperuricemia (*p* for overall < 0.001, *p* for nonlinearity = 0.0013, Figure 3). Figure 3 also revealed that within this specified intake range, there is a notable decrease in the risk of hyperuricemia as legume intake increases. This observation suggests a threshold effect, where consuming between 6 and 28 g of legumes per day may be particularly beneficial in lowering the risk of hyperuricemia.

## 4. Discussion

In this large-scale prospective cohort study involving 43,371 adult residents in suburban Shanghai, China, we observed an inverse nonlinear association between legume intake and the risk of incident hyperuricemia, with the most pronounced risk reduction observed at a daily intake range of 6–28 g. This association remained robust after comprehensive adjustment for sociodemographic, lifestyle, and physiological information, and was consistent across sensitivity and subgroup analyses.

The relationship between diet and hyperuricemia has garnered substantial attention, as diet—an important modifiable behavior—plays a crucial role in the prevention of hyperuricemia [35,36]. In the urbanization process of developing countries represented by China, the shift in food consumption profiles from traditional staple foods to animal-derived foods has become increasingly recognized. One study projected future food landscapes under 2030 urbanization scenarios, revealing that among all food categories surveyed, animal-derived foods—including red meat in particular—showed pronounced upward trends in both production and consumption [37]. However, certain food components, such as red meat, are recognized as pro-inflammatory stimuli [38].

Recent studies have indicated that a pro-inflammatory diet promotes the development of hyperuricemia [39]. Legumes, however, are essential for human nutrition: they are recognized as low-sodium, high-potassium foods that can effectively inhibit the renin-angiotensin system, reduce vasoconstriction, and thereby contribute to blood pressure reduction [40]. Additionally, legumes are rich in proteins, folic acid, flavonoid bioactive compounds, and various micronutrients such as iron, zinc, calcium, and magnesium, exerting antioxidant and anti-inflammatory effects [41,42]. The potential mechanisms underlying the beneficial effects of legumes may involve the following aspects. First, isoflavones possess a molecular structure analogous to estrogen, enabling them to bind to estrogen receptors and exert estrogen-like effects that contribute to uric acid inhibition under specific conditions [14]. Second, xanthine oxidase serves as the key enzyme catalyzing the conversion of xanthine and hypoxanthine to uric acid. Structure–activity investigations have demonstrated that the -OH groups and their substitution patterns on the benzene ring of flavonoids are critical for xanthine oxidase inhibition. Furthermore, molecular docking studies have shown that flavonoids prevent uric acid formation by interacting with xanthine oxidase substrates via hydrophobic, hydrogen bonds, and π-π interaction [43]. Third, folic acid can inactivate xanthine oxidoreductase enzyme, the enzyme responsible for the sequential oxidation of hypoxanthine to xanthine and xanthine to uric acid. In particular, folate inhibits xanthine oxidoreductase enzyme via high-affinity slow binding at the molybdenum site—the purine-interacting domain—thereby disrupting the interaction of the enzyme with xanthine and hypoxanthine [44]. Previous studies have shown that legumes may confer beneficial impacts on SUA levels [45]. The findings of the present study support this notion and are consistent with those of previous relevant studies [46,47,48].

Notably, the protective association between legume intake and reduced risk of hyperuricemia was observed even when legume consumption was below the recommended nutrient intake (RNI). The RNI is formulated based on the estimated average requirement and serves to evaluate the adequacy of individual nutrient intake. It represents a population-level target designed to meet the needs of nearly all healthy people, rather than a threshold below which health benefits are absent. Modest legume intake, even at sub-recommended levels, still delivers beneficial bioactive components. Relative to minimal or no legume intake, moderate consumption may still confer measurable protection against hyperuricemia. This finding carries important public health implications, suggesting that individuals do not need to immediately reach recommended intake levels to obtain health benefits. Promoting gradual increases in legume consumption may help reverse the current low intake status in the adult population and facilitate the primary prevention of hyperuricemia.

Furthermore, the results of this study revealed that the median legume intake among adult residents in suburban Shanghai was only 5.57 g/d, which will not only compromise individual health, but also pose a challenge to the development goal of a sustainable diet [49], which is far from the recommended range of China Dietary Guidelines (2022) [50]. However, a previous cross-sectional study reported inconsistent findings with ours [51]. This discrepancy may be attributed to the direct conversion of soybean milk consumption to legume intake in that study, leading to an overestimation of actual legume intake. Additionally, differences in the food composition of legume intake and cooking practices across regions may have contributed to the inconsistent results. To our knowledge, prospective studies investigating the association between legume intake and hyperuricemia remain limited in China.

To our knowledge, this is the first prospective cohort study to examine the dose–response association between legume intake and hyperuricemia incidence among adults in suburban Shanghai, a highly urbanized coastal metropolis facing a dual burden of escalating hyperuricemia prevalence and rapid dietary transition. By leveraging large-scale prospective cohort data with comprehensive biobank resources from suburban Shanghai, our findings fill a critical evidence gap where previous research was limited to a single cross-sectional study of adult males [52]. The robust inverse nonlinear association, which persisted after extensive adjustment for sociodemographic, lifestyle, and physiological confounders, provides novel causal insights that cross-sectional designs cannot establish. Furthermore, the identification of a specific optimal intake range offers precise, actionable evidence for developing tailored dietary interventions in urban Chinese adult populations, especially coastal cities.

Global Food-Based Dietary Guidelines have indicated that legumes are safe and beneficial when consumed within a reasonable range [53], which is consistent with the findings of our study. From the viewpoint of balanced nutrition and health promotion, increasing legume intake not only helps reduce the risk of hyperuricemia but also provides abundant nutritional support for the human body, aligning with the balanced diet concept advocated by the Chinese Dietary Guidelines (2022) [50]. However, globally, especially in East and Southeast Asia, legume intake is below the recommended level and has declined significantly [54]. According to the standards of the Chinese Dietary Guidelines, the proportion of adults aged 55 years and above with insufficient legume intake in China is as high as 74%, indicating a severe inadequacy [55], which may be related to traditional dietary habits, food availability, economic factors, and other aspects [56].

Legume cultivation has a relatively low environmental impact; therefore, effective strategies are needed to increase legume consumption for the benefit of human and planetary health [57]. First, public awareness and acceptance of legumes can be enhanced through culinary and educational initiatives [58]. Relevant departments and organizations can provide legume-based recipes and cooking courses to help consumers better integrate legumes into their daily diets [59] and further play their role as a sustainable and healthy nutritional substitute to meat [3]. Second, cultural and social factors play an important role in legume consumption, and support from family and social environments has a positive impact [60]. Additionally, policy-level support and promotion are crucial [61] to advance health and sustainable development goals [62]. Future studies can further explore the associations between legume intake and various health outcomes, particularly differences across populations and regions [63,64].

The strengths of this study include its community-based, large-sample prospective cohort design. To our knowledge, this is the largest prospective cohort study to date investigating the relationship between legume intake and hyperuricemia among adults in a rapidly urbanizing coastal region of China. The study’s advantages also include large-scale population sampling, comprehensive adjustment for confounders, and rigorous quality control. However, this study also has several limitations. First, legume intake was assessed using the FFQ, and recall bias is inevitable. Additionally, since the intake of each type of legume was not recorded separately, it was not possible to identify the specific legume type(s) contributing to the reduced risk of hyperuricemia. Future research needs to explore the impact of individual legume types and their products. Nevertheless, most of the legumes involved in this study share similar nutritional profiles and bioactive components. Therefore, total legume intake can reasonably represent the cumulative beneficial effect of this food group, even in the absence of specific legume type data. This overall pattern may still provide meaningful population-level evidence for dietary recommendations in the prevention of hyperuricemia. Second, the specific timing of legume consumption (e.g., meal times) was not documented, and consuming certain foods at specific times of the day may be associated with a lower risk of hyperuricemia [65]. Third, the study did not include data on participants’ dietary supplement intake. Fourth, residual reverse causality cannot be completely ruled out—individuals with health awareness may increase legume intake after developing subclinical symptoms. Finally, all participants were from the coastal city of Shanghai in East China, and the generalizability of this finding to other populations requires further verification.

## 5. Conclusions

In conclusion, legume intake is nonlinearly and inversely associated with the risk of incident hyperuricemia among the adult residents in Shanghai suburbs located in coastal cities, with the most significant risk reduction at a daily intake of 6–28 g; notably, even modest consumption (below the RNI) confers protective effects. Since the median daily legume intake of Shanghai suburban adults (5.57 g) is far below the China Dietary Guidelines (2022) recommendations, comprehensive strategies—including health education, policy support, and cultural promotion—are recommended to guide individuals in increasing legume consumption and adjusting dietary structures for hyperuricemia prevention.

## Figures and Tables

**Table 1:** Baseline characteristics of participants according to the categories of the intake of legumes.

Characteristics	Q1 (N = 12,917)	Q2 (N = 17,602)	Q3 (N = 12,852)	Total (N = 43,371)
Incidence of density ***	7.94 (7.28, 8.59)	5.83 (5.34, 6.33)	5.79 (5.20, 6.38)	6.47 (6.14, 6.80)
Gender (%) ***	4018 (31.11)	6279 (35.67)	5381 (41.87)	15,678 (36.15)
Age (year) ***	59 (51, 65)	59 (51, 65)	57 (47, 65)	58 (50, 65)
Age group ***				
20–39	1248 (9.66)	1627 (9.24)	2015 (15.68)	4890 (11.28)
40–49	1539 (11.92)	2111 (11.99)	1903 (14.81)	5553 (12.80)
50–59	4192 (32.45)	5694 (32.35)	3520 (27.39)	13,406 (30.91)
60–69	4713 (36.49)	6544 (37.18)	4243 (33.01)	15,500 (35.74)
70–74	1225 (9.48)	1626 (9.24)	1171 (9.11)	4022 (9.27)
Educational level (%) ***				
Primary school or below	5259 (40.71)	5946 (33.78)	2551 (19.85)	13,756 (31.72)
Junior high school	4820 (37.32)	7158 (40.67)	4966 (38.64)	16,944 (39.07)
Senior high school or above	2838 (21.97)	4498 (25.55)	5335 (41.51)	12,671 (29.21)
Marital status (%) ***				
Unmarried	220 (1.70)	237 (1.35)	372 (2.90)	829 (1.91)
Married	11,713 (90.68)	16,369 (92.99)	11,670 (90.80)	39,752 (91.66)
Divorced and other	984 (7.62)	996 (5.66)	810 (6.30)	2790 (6.43)
Retirement (%) ***	8118 (62.85)	11,116 (63.15)	7208 (56.08)	26,442 (60.97)
Smoking (%) ***	2275 (17.61)	3534 (20.08)	2821 (21.95)	8630 (19.90)
Alcohol drinking (%) ***	1159 (8.97)	1866 (10.60)	1486 (11.56)	4511 (10.40)
PA level (%) ***				
Low	8062 (62.41)	10,174 (57.80)	6502 (50.59)	24,738 (57.04)
Moderate	3779 (29.26)	5892 (33.47)	4781 (37.20)	14,452 (33.32)
High	1076 (8.33)	1536 (8.73)	1569 (12.21)	4181 (9.64)
Sleep time (%) ***				
<5 h	753 (5.83)	769 (4.37)	530 (4.12)	2052 (4.73)
5–8 h	9467 (73.29)	13,727 (77.98)	10,220 (79.52)	33,414 (77.04)
≥8 h	2697 (20.88)	3106 (17.65)	2102 (16.36)	7905 (18.23)
BMI group (%)				
<18.5 kg/m^2^	443 (3.43)	565 (3.21)	443 (3.45)	1451 (3.35)
18.5–23.9 kg/m^2^	6333 (49.03)	8683 (49.33)	6492 (50.51)	21,508 (49.59)
24.0–27.9 kg/m^2^	4765 (36.89)	6522 (37.05)	4619 (35.94)	15,906 (36.67)
≥28.0 kg/m^2^	1376 (10.65)	1832 (10.41)	1298 (10.10)	4506 (10.39)
WC (cm)	81.00 (74.50, 87.00)	81.00 (74.67, 87.00)	81.00 (74.75, 87.50)	81.00 (74.67, 87.00)
Energy intake (kcal/day) ***	1023.20 (833.79, 1324.69)	1075.16 (883.43, 1342.86)	1397.85 (1114.23, 1789.55)	1142.76 (909.38, 1482.56)
Cereals and potatoes (g/day) ***	351.42 (256.57, 498.87)	347.82 (250.52, 473.83)	347.82 (242.85, 466.43)	347.16 (250.00, 478.57)
Vegetables (g/day) ***	200.00 (100.00, 300.00)	200.00 (100.00, 300.00)	200.00 (100.00, 300.00)	200.00 (100.00, 300.00)
Mushrooms (g/day) ***	11.43 (3.29, 28.57)	14.29 (6.58, 28.57)	28.57 (14.29, 33.14)	14.29 (6.58, 28.57)
Fruits (g/day) ***	100.00 (28.57, 150.00)	100.00 (28.57, 150.00)	100.00 (50.00, 200.00)	100.00 (40.00, 150.00)
Milk and dairy products (g/day) ***	38.57 (0.00, 189.29)	50.00 (0.00, 178.57)	85.71 (6.08, 240.00)	63.23 (0.00, 200.00)
Red meat (g/day) ***	33.24 (17.57, 57.14)	35.15 (18.23, 57.14)	53.29 (28.57, 85.71)	35.15 (20.86, 64.29)
White meat (g/day) ***	54.87 (29.59, 90.65)	67.01 (45.01, 100.00)	95.58 (63.72, 138.43)	70.29 (42.86, 114.29)
Eggs (g/day) ***	27.50 (15.71, 55.00)	28.57 (15.71, 55.00)	50.00 (15.71, 55.00)	31.43 (15.71, 55.00)
Processed meats (g/day) ***	0.00 (0.00, 1.97)	0.00 (0.00, 3.29)	0.49 (0.00, 5.46)	0.00 (0.00, 3.29)
Nuts (g/day) ***	3.29 (0.33, 14.29)	6.58 (1.32, 14.29)	8.57 (1.64, 28.57)	6.58 (0.82, 15.00)
Legumes (g/day) ***	0.90 (0.21, 1.76)	5.57 (3.71, 7.43)	11.14 (8.68, 15.45)	5.57 (2.28, 7.99)
History of chronic diseases (%)				
Hypertension ***	6316 (48.9)	8598 (48.85)	5739 (44.65)	20,653 (47.62)
CHD	591 (4.58)	781 (4.44)	593 (4.61)	1965 (4.53)
Diabetes	1883 (14.58)	2609 (14.82)	1916 (14.91)	6408 (14.77)
Dyslipidemia	4292 (33.23)	5912 (33.59)	4444 (34.58)	14,648 (33.77)
Chronic bronchitis **	967 (7.49)	1169 (6.64)	821 (6.39)	2957 (6.82)
Asthma *	347 (2.69)	387 (2.20)	292 (2.27)	1026 (2.37)
COPD	78 (0.60)	89 (0.51)	72 (0.56)	239 (0.55)

The median (inter-quartile range) was displayed for non-normal continuous variables, and the mean (SD) was displayed for normal continuous variables. Categorical variables were displayed using frequency (represented as percentages). The Kruskal–Wallis rank test or ANOVA was used for continuous variables and the Mantel–Haenszel χ^2^ test was used for categorical variables. Abbreviations: PA, physical activities; BMI, body mass index; WC, waist circumference; CHD, coronary heart disease; COPD, chronic obstructive pulmonary disease; SD, standard deviation; ANOVA, analysis of variance. *** *p* < 0.001, ** *p* < 0.01, * *p* < 0.05.

**Table 2:** Hazard ratios (95% *CIs*) of hyperuricemia by the intake of legume groups.

	Per-Unit Increase in the Intake of Legumes	Legumes Intake Group			*p* for Trend ^a^
		Q1	Q2	Q3	
Incidence of Density (*per 1000 person-years*)	6.47	7.94	5.83	5.79	—
No. of cases	1456	552	539	365	—
Follow-up person-years	225,002.40	69,564.51	92,384.14	63,053.75	—
Model 1 ^b^	0.98 (0.97, 0.99) *	1.00	0.73 (0.65, 0.82) *	0.78 (0.68, 0.89) *	<0.001
Model 2 ^b^	0.98 (0.97, 0.99) *	1.00	0.72 (0.64, 0.82) *	0.74 (0.64, 0.85) *	<0.001
Model 3 ^b^	0.98 (0.97, 0.99) *	1.00	0.73 (0.65, 0.82) *	0.74 (0.64, 0.86) *	<0.001

^a^ A linear trend was calculated by treating median values across three categories as a continuous variable in the model. ^b^ Hazard ratios (95% *CIs*) of the intake of legumes with hyperuricemia were examined using Cox proportional-hazards regression models; Model 1 was adjusted for sociological demographic characteristics (age, gender, educational levels, marital status and retirement). Model 2 was adjusted further for lifestyle information (smoking, alcohol drinking, PA levels, sleep time, BMI, WC, dietary energy intakes, consumption of cereals and potatoes, vegetables, mushrooms, fruits, red meat, white meat, milk and dairy products, eggs, and processed meats). Model 3 was adjusted further for the physiological information (hypertension, CHD, diabetes, dyslipidemia, COPD, chronic bronchitis and asthma). Abbreviations: PA, physical activity; BMI, body mass index; WC, waist circumference; CHD, coronary heart disease; COPD, chronic obstructive pulmonary disease; CIs, confidence intervals. * *p* < 0.05.

**Table 3:** Sensitivity analysis of hazard ratios (95% *CIs*) of hyperuricemia by three categories of the intake of legumes.

Sensitivity	Per-Unit Increase in the Intake of Legumes	Legumes Intake Group			*p* for Trend ^a^
		Q1	Q2	Q3	
Exclusion 1 (n = 43,247)	0.98 (0.97, 0.99) *	1.00	0.73 (0.64, 0.83) *	0.74 (0.64, 0.87) *	<0.001
Exclusion 2 (n = 43,010)	0.98 (0.97, 0.99) *	1.00	0.71 (0.62, 0.82) *	0.75 (0.64, 0.89) *	<0.001
Exclusion 3 (n = 38,481)	0.98 (0.97, 0.99) *	1.00	0.73 (0.65, 0.83) *	0.75 (0.65, 0.87) *	<0.001

^a^ A linear trend was calculated by treating median values across three categories as a continuous variable in the model. The model was adjusted for age, gender, educational levels, marital status, retirement, smoking, alcohol drinking, PA levels, sleep time, BMI, WC, dietary energy intakes, consumption of cereals and potatoes, vegetables, mushrooms, fruits, red meat, white meat, milk and dairy products, eggs, processed meats, and physiological information (hypertension, CHD, diabetes, dyslipidemia, COPD, chronic bronchitis and asthma). Exclusion 1 excluded hyperuricemia cases diagnosed within the first year of the study’s observation period (n = 124). Exclusion 2 excluded hyperuricemia cases diagnosed within the first two years of the study’s observation period (n = 361). Exclusion 3 excluded participants under 40 years old at the baseline survey (n = 4890). Abbreviations: PA, physical activity; BMI, body mass index; WC, waist circumference; CHD, coronary heart disease; COPD, chronic obstructive pulmonary disease; CIs, confidence intervals. * *p* < 0.05.

## Data Availability

The datasets generated and/or analyzed during the current study are not publicly available due to licensing restrictions and data use agreements. Qualified researchers may request access to these data from the author (Genming Zhao) with appropriate justification and pending approval from the data custodian.

## Abbreviations

The following abbreviations are used in this manuscript:

GI	Glycemic Index
SUA	Serum Uric Acid
SSACB	Shanghai Suburban Adult Cohort and Biobank
PA	Physical Activity
WC	Waist Circumference
EMR	Electronic Medical Record System
CDSS	Cause-of-Death Surveillance System
ICD-10	International Classification of Diseases, 10th Revision
CKD	Chronic Kidney Disease
FFQ	Food Frequency Questionnaire
BMI	Body Mass Index
HbA1C	Glycosylated Hemoglobin A1C
FPG	Fasting Plasma Glucose
CHD	Coronary Heart Disease
COPD	Chronic Obstructive Pulmonary Disease
SD	Standard Deviation
ANOVA	Analysis of Variance
IQR	Interquartile Range
HR	Hazard Ratio
CIs	Confidence Intervals
RCSs	Restricted Cubic Splines
STROBE	Strengthening the Reporting of Observational Studies in Epidemiology
RNI	Recommended Nutrient Intake
